# University Students' Satisfaction with their Academic Studies: Personality and Motivation Matter

**DOI:** 10.3389/fpsyg.2016.00055

**Published:** 2016-02-16

**Authors:** F.-Sophie Wach, Julia Karbach, Stephanie Ruffing, Roland Brünken, Frank M. Spinath

**Affiliations:** ^1^Department of Psychology, Saarland UniversitySaarbrücken, Germany; ^2^Department of Educational Science, Saarland UniversitySaarbrücken, Germany; ^3^Department of Psychology, Johann Wolfgang Goethe-Universität Frankfurt am MainFrankfurt am Main, Germany

**Keywords:** satisfaction with academic studies, student satisfaction, personality, motivation, achievement, intelligence

## Abstract

Although there is consensus about the importance of students' satisfaction with their academic studies as one facet of academic success, little is known about the determinants of this significant outcome variable. Past research rarely investigated the predictive power of multiple predictors simultaneously. Hence, we examined how demographic variables, personality, cognitive and achievement-related variables (intelligence, academic achievement), as well as various motivational constructs were associated with three different dimensions of satisfaction (satisfaction with study content, satisfaction with the conditions of the academic program, satisfaction with the ability to cope with academic stress) assessed approximately 2 years apart. Analyzing data of a sample of university students (*N* = 620; *M*_age_ = 20.77; *SD*_age_ = 3.22) using structural equation modeling, our results underline the significance of personality and motivational variables: Neuroticism predicted satisfaction with academic studies, but its relevance varied between outcome dimensions. Regarding the predictive validity of motivational variables, the initial motivation for enrolling in a particular major was correlated with two dimensions of subsequent satisfaction with academic studies. In contrast, the predictive value of cognitive and achievement-related variables was relatively low, with academic achievement only related to satisfaction with the conditions of the academic program after controlling for the prior satisfaction level.

## Introduction

Whereas extensive research has been conducted on employees' satisfaction with work, less is known about a construct of similar importance for students' lives: students' satisfaction with their academic studies (SAS). SAS is considered to be an important subjective educational outcome variable due to its relation to a wide range of crucial constructs (Benjamin and Hollings, 1997), such as stress tolerance (Schiefele and Jacob-Ebbinghaus, 2006), retention (Starr et al., 1972), and academic achievement (Bean and Bradley, 1986). Nevertheless, the significance of SAS as a subjective indicator of academic success (Spörer and Brunstein, 2005) has often been neglected (Trapmann et al., 2007). Since the competition among universities for high achieving students has increased and it is a political as well as a social objective to improve the likelihood of students' persistence in their studies, the concept of SAS and especially its antecedents is gaining importance. As a consequence, this study aims at identifying the prerequisites of becoming a highly satisfied student, which in turn might stimulate modifications in university environments, support students' adjustments, lead to higher performance levels, and prevent students from dropping out (Starr et al., 1971; Wiers-Jenssen et al., 2002). However, despite the significance of SAS, its research area has only “few theoretical underpinnings” (p. 213; Benjamin and Hollings, 1997) and lacks a generally accepted definition of the construct (Apenburg, 1980; Benjamin and Hollings, 1997). Some authors, for instance, emphasized cognitive (e.g., Okun and Weir, 1990) or affective aspects of SAS (e.g., Reed et al., 1984; Bean and Bradley, 1986), whereas Westermann et al. (1996) described SAS as someone's attitude toward their academic studies. Many authors characterize and define SAS in analogy to job satisfaction (Apenburg, 1980; Westermann et al., 1996; Trapmann et al., 2007) capitalizing on major similarities between study and work conditions such as being the major activity during the day, having a similar urge of achieving goals and meeting certain requirements, as well as providing the opportunity of satisfying additional needs, e.g., self-actualization (Starr et al., 1972; Apenburg, 1980). Accordingly, we also refer to studies on job satisfaction when presenting the current state of research. The lack of both a commonly accepted definition and an established theoretical framework of the construct has also implications on the operationalization of SAS, culminating in the complaint that “there are almost as many different measures of satisfaction reported as there are studies investigating the construct” (p. 68, Reed et al., 1984). Furthermore, many of these instruments focus on the evaluation of external circumstances (e.g., housing, tuition). However, these external circumstances also differ significantly between countries and, hence, several instruments may not apply to different educational systems, which is maybe why often new instruments had to be developed. Moreover, some studies operationalized SAS by single-item scales, which have been criticized for their psychometric disadvantages (see Diener, 1984; for an overview).

Fortunately, with the *study-satisfaction questionnaire* (Westermann et al., 1996; Schiefele and Jacob-Ebbinghaus, 2006; see Section Satisfaction with Academic Studies and Global Life Satisfaction) an instrument exists that has been frequently used to assess SAS (e.g., Heise et al., 1997; Hiemisch et al., 2005; Schiefele and Jacob-Ebbinghaus, 2006) and that focuses to a greater extent on internal evaluation processes, making generalizability to other educational systems more likely. Additionally, it takes the assumed complex and multidimensional structure of the construct (Benjamin and Hollings, 1997; Wiers-Jenssen et al., 2002) into account by differentiating three dimensions of SAS: *Satisfaction with study content* (*SAS-Content*) reflects the student's feelings of joy and satisfaction regarding his or her chosen major. *Satisfaction with the terms and conditions of the academic program* (*SAS-Conditions*) provides information on how the student experiences the environment at his or her university. Finally, *satisfaction with the personal ability to cope with academic stress* (*SAS-Coping*) captures the impact of academic stress on students' personal lives.

Given that SAS is a complex phenomenon (Benjamin and Hollings, 1997) a considerable variety of variables has to be examined in order to identify valid predictors of SAS.

Investigating the predictive validity of cognitive and achievement-related variables as well as motivational variables for SAS is particularly important, given that SAS can be considered one facet of academic success. Previous studies focusing on the relationship between academic achievement and SAS found that students' grade point average was moderately associated with SAS (*r* = 0.35; Nauta, 2007). Regarding the causal relationship between achievement and SAS, Apenburg (1980) reported larger correlations between achievement and subsequent SAS than vice versa. However, although the relationship between grades and SAS has repeatedly been demonstrated, it seems to be just one among many variables associated with SAS (Howard and Maxwell, 1980). For instance, the importance of more subjective performance measures must be taken into account, because it has been shown that students who evaluated their own achievement more positively tended to report higher levels of SAS (Wiers-Jenssen et al., 2002). A large number of studies examined the significance of different motivational constructs for students' SAS. For instance, a study by Kaub et al. (2012) demonstrated the relevance of students' vocational interests for their level of SAS. Moreover, students expressing a rather intrinsic motivation for choosing a certain major were also more satisfied with their academic studies, whereas extrinsic motivation was not associated with SAS (Künsting and Lipowsky, 2011). These results partly support findings by Heise et al. (1997) who demonstrated the relevance of intrinsic motivation especially for SAS-Content (see also Schiefele and Jacob-Ebbinghaus, 2006). Furthermore, the use of a self-regulated learning-style predicted SAS (Spörer and Brunstein, 2005).

In addition, the relationship between personality traits and SAS has been investigated. Referring to the five-factor model of personality, neuroticism consistently showed negative relations to SAS (Trapmann et al., 2007; Künsting and Lipowsky, 2011). A meta-analysis focusing on the five factors of personality and several criteria for academic success at the university level reported an average corrected correlation of −0.369 for the association between neuroticism and SAS (Trapmann et al., 2007). However, the study included only eight independent samples, indicating how little attention the research field of SAS has received so far. In the area of job satisfaction, a meta-analysis by Judge et al. (2002) reported a similar association for neuroticism (ρ = −0.29), which made it “the strongest and most consistent correlate of job satisfaction” (p. 534), followed by conscientiousness and extraversion. In contrast, agreeableness and openness to experience showed rather weak associations with job satisfaction.

Given the lack of a commonly accepted definition of SAS, the comparatively small number of studies on potential predictor variables of SAS, and the absence of replications of these isolated findings, the field is still characterized by inconsistent results and restricted comparability (see also Reed et al., 1984). Additionally, most of the existing research investigated the predictive validity of few selected variables in cross-sectional designs, not considering the possibility of SAS being influenced by several constructs. Hence, the present study extended the focus of previous research by *simultaneously* examining the predictive validity of achievement, motivation, and personality for subsequent SAS. Moreover, most of previous research did not take the multidimensional structure of SAS into account by operationalizing SAS in a domain-general manner. Thus, it remained unclear whether the predictive value of the constructs under study would differ between the three SAS-dimensions (SAS-Content, SAS-Conditions, and SAS-Coping). We expected domain-general *and* domain-specific effects: Concerning the importance of personality traits, students scoring higher on neuroticism should score lower on all SAS dimensions (hypothesis 1; Trapmann et al., 2007; Künsting and Lipowsky, 2011). In contrast, we expected positive relations between SAS and both conscientiousness (hypothesis 2) and extraversion (hypothesis 3), based on the findings on job satisfaction (Judge et al., 2002). Since the subscale SAS-Coping did not only take the personal stress level within the academic program into account, but also provided information as to how this stress interfered with the participants' personal lives, SAS-Coping covered a wider area of the individuals' experiences than SAS-Content or SAS-Conditions. As a consequence, we expected that this would be reflected in a greater contribution of trait variables like personality to SAS-Coping than to SAS-Content or SAS-Conditions (hypothesis 4). Furthermore, we hypothesized that cognitive and achievement-related (hypothesis 5; Apenburg, 1980; Nauta, 2007) as well as motivational variables (hypothesis 6; Heise et al., 1997; Spörer and Brunstein, 2005; Künsting and Lipowsky, 2011; Kaub et al., 2012) were more strongly associated with SAS-Content and SAS-Conditions, which focused merely on the academic program. Moreover, we were interested in investigating whether these variables would also be associated with subsequent SAS dimensions, after prior SAS has been taken into account. This issue has not been examined so far, hence this research question has to be considered as exploratory (research question 1).

It is important to know that SAS as well as job satisfaction are considerably related to global life satisfaction (Tait et al., 1989; Lounsbury et al., 2005). However, since our work focuses on SAS, we were particularly interested in variables that predicted this specific facet of satisfaction. Hence, we controlled for global life satisfaction to ensure that correlations between SAS and its predictors were not merely based on underlying associations between the respective predictors and global life satisfaction.

## Materials and methods

### Participants and procedure

We investigated the predictive validity of multiple predictor variables on students' subsequent SAS, using data derived from the “Study on the Impact of Individual and Organizational Variables on Academic Achievement in Teacher Education (SioS-L),” conducted in Germany between 2009 and 2015. A total of 620 (age: ranging from 17 to 44, *M* = 20.77, *SD* = 3.22; sex: female *n* = 389; male: *n* = 231) first-year students majoring in teacher education completed a battery of tests at the beginning of their studies, including measures of motivational aspects (vocational interests, academic self-concept, self-regulation, achievement motivation, motivation for choosing teacher education), personality, intelligence, and demographic information (age, sex, their parents' highest academic degree) as well as their SAS at that time. A total of 255 students (41.1% of the original sample; 5.4% missing values in the complete dataset) agreed to participate in a second investigation on average 2 years and 2 months after the first assessment (intervals ranging from 1 year and 8 months to 2 years and 7 months). The second assessment comprised self-reports on the participants' global life satisfaction and again their SAS. Participants' grades in two educational exams were obtained from the examination office between the two assessments. The students were informed that all data were kept confidential. Their participation was voluntarily and all participants provided informed consent. Furthermore, the research project was approved by the ethics committee of the German Psychological Society. Students received course credit points or payment for their participation.

### Measures

#### Satisfaction with academic studies and global life satisfaction

Students' SAS both at the beginning of the participants' studies and approximately 2 years later was measured by means of the *study-satisfaction questionnaire* (Westermann et al., 1996; Schiefele and Jacob-Ebbinghaus, 2006), consisting of 10 items representing the three SAS dimensions. The participants were asked to rate on a five-point scale (ranging from 1 = “*strongly agree”* to 5 = “*strongly disagree”*) to what extent they agreed with statements related to the area of SAS-Content (e.g., “I really enjoy the subject of my studies.”), SAS-Conditions (e.g., “I wish the study conditions at my university were better.”), and SAS-Coping (e.g., “I am not able to reconcile my study requirements with other personal obligations.”). If necessary, items were recoded, so that higher values always indicated higher SAS. Manifest correlations between the two assessments of SAS ranged from 0.38 (SAS-Content) to 0.42 (SAS-Coping, all *p*'s < 0.001) and were therefore similar to stability scores reported for job satisfaction (see Dormann and Zapf, 2001). Internal consistencies of raw scores ranged from Cronbach's α = 0.78 (wave 1 SAS-Conditions) to 0.87 (wave 2 SAS-Content). The three-dimensional structure of the questionnaire has been confirmed in different samples (Westermann et al., 1996; Schiefele and Jacob-Ebbinghaus, 2006).

Furthermore, in order to control for global life satisfaction, participants' completed the German version of the *satisfaction with life scale* (SWLS; Schumacher, 2003) as part of the second assessment. The seven-point scale ranging from 1 (“*totally agree”*) to 7 (“*totally disagree”*) was recoded as well so that higher values represented higher levels of life satisfaction (Cronbach's α [calculated based on raw data] = 0.89). The SWLS is widely used to assess life satisfaction. Its factorial validity has also been confirmed for the German version (Glaesmer et al., 2011).

#### Demographic information

Within the set of questionnaires applied at the first assessment, students provided their date of birth, their sex, and information on their parents' academic degree. Specifically, they indicated for both parents separately whether they graduated from 1 = a vocational secondary school (German “Hauptschule”/“Volksschule”), 2 = from a middle track secondary school (German “Realschule”), 3 = received general permission to attend university (German “Abitur”), or 4 = graduated from a university. The higher of the two parental academic degrees served as an indicator of the participant's educational background.

#### Cognitive and achievement-related variables

Intelligence was assessed by a short version of Horn's German *performance test system* (German “Leistungsprüfsystem”, LPS; Horn, 1983), comprising eight timed subtests (verbal comprehension, word fluency, word comprehension, spatial visualization, reasoning, perceptual speed, number facility). Each item solved correctly received a score of one and the majority of subscales consisted of 40 items. We calculated a total score (mean of the eight subtest scores; Cronbach's α [calculated based on raw data] = 0.72), which served as an indicator of the participant's intelligence. Information on the validity of the instrument has been provided elsewhere (see in detail Horn, 1983).

Individual's academic achievement was measured by the participants' averaged grades (ranging from 1 = “*outstanding”* to 5 = “*failed”*) in two written exams from the core curriculum of educational science.

#### Motivational variables

We used the revised *general interest structure test* (German “Allgemeiner Interessen-Struktur-Test—Revision”, AIST-R; Bergmann and Eder, 2005) to measure six different vocational interests at the beginning of the participants' studies (realistic, investigative, artistic, social, enterprising, conventional; Holland, 1997). The students rated on a five-point scale (ranging from 1 = “*very interested”* to 5 = “*not interested at all”*) to what extent they were interested in typical activities of each dimension. We found that the questionnaire is a reliable assessment of vocational interests (Cronbach's α [calculated based on raw data] ranging from 0.79 to 0.86) and its factorial validity as well as associations to other instruments assessing interests has already been demonstrated (Bergmann and Eder, 2005).

Moreover, the students completed the *academic self-concept scales* (Dickhäuser et al., 2002) in the first assessment. All four scales of the instrument allowed the participants to evaluate their own academic abilities on a seven-point semantic differential. These scales differ regarding their reference norms: Three scales assess the participants' academic competencies in comparison to a social, individual, and a criterion-oriented reference norm, whereas the last scale determines how the participants evaluate their abilities independently of any comparison group. We calculated a composite score (mean of the four subscales) and reliability analysis conducted with raw data revealed a good internal consistency (Cronbach's α = 0.85). Information on convergent and discriminant validity of the instrument has been reported (Dickhäuser et al., 2002).

In order to assess students' initial achievement motivation, we applied a short version of the German *achievement motivation inventory* (German “Leistungsmotivationsinventar”, LMI-K; Schuler and Prochaska, 2001). The 30 items were rated on a seven-point scale, ranging from 1 (“*completely true”*) to 7 (“*not true at all”*). The short version showed a good internal consistency in our sample (Cronbach's α [calculated based on raw data] = 0.92) and the long version has been validated against various criteria (Schuler and Prochaska, 2001).

We used the *self-regulation scale* (German “Selbstregulation”, REG; Schwarzer, 1999) to measure the participants' self-regulation at the beginning of their studies. The students were asked to rate on a four-point scale (ranging from 1 = “*completely true”* to 4 = “*not true at all”*) to what extent they agreed with 10 different statements. Reliability analyses conducted with raw data suggested a satisfactory internal consistency of the scale (Cronbach's α = 0.78).

The German *motivations for choosing teacher education questionnaire* (German “Fragebogen zur Erfassung der Motivation für die Wahl des Lehramtsstudiums”, FEMOLA; Pohlmann and Möller, 2010) was used to operationalize the participants' intrinsic and extrinsic motivation for enrolling in teacher education. It included six different subscales (intrinsic motivation: educational interest, subject-specific interest, ability beliefs; extrinsic motivation: utility, social influences, low difficulty of the study). This construct is of particular relevance in a German sample, since the occupational group of teachers in general has a high reputation within the German population. Moreover, most teachers are employed by the state and receive a comparatively high salary. As a consequence, motivation for freshmen to enroll in teacher education can vary substantially. All items in this questionnaire were rated on a four-point scale, ranging from 1 (“*completely true”*) to 4 (“*not true at all”*). Internal consistencies of raw scores varied between Cronbach's α = 0.71 and 0.90 within our sample. The validity of the instrument has been demonstrated by the authors (Pohlmann and Möller, 2010).

Where necessary, variables were recoded so that higher values consistently represented higher scores on the respective motivational variables.

#### Personality variables

The students' five factors of personality were assessed at the beginning of their studies by means of the widely used German version of the *NEO five-factor-inventory* (German “NEO-Fünf-Faktoren-Inventar”, NEO-FFI; Borkenau and Ostendorf, 2008). The participants were asked to rate on a five-point scale (ranging from 1 = “*strongly agree”* to 5 = “*strongly disagree”*) to what extent they agreed with certain statements. Again, variables were recoded so that higher values indicated a more pronounced personality trait. Internal consistencies of raw scores varied between Cronach's α = 0.73 and 0.86 within our sample. It has been demonstrated that the instrument also provides a valid assessment of the five personality traits neuroticism, extraversion, openness to experience, agreeableness, and conscientiousness (Borkenau and Ostendorf, 2008).

### Data analysis

We examined the differential predictive validity of the described variables (sex, parental academic degree, intelligence, grades in educational exams, motivational variables, and personality) assessed at the beginning of the participants' studies for the three SAS dimensions (SAS-Content, SAS-Conditions, and SAS-Coping) approximately 2 years later, using a structural equation modeling approach. In order to control for the large age range within our sample, age was partialled from predictor and criterion variables. In addition, we controlled for global life satisfaction in the criterion variables in a comparable manner. Hence, all statistical analyses were then conducted with residualized scores. This way we ensured that any effects on SAS did not simply occur due to underlying associations with age or global life satisfaction (see also Section Introduction). We specified one prediction model for each SAS dimension. For the sake of parsimony, only variables showing a significant manifest relationship with the criterion in prior correlational analyses were included into the model. Since this approach led to relatively large models, either subtest scores or parcels were used as indicators of the latent construct if this latent construct had otherwise comprised more than four items as indicators. We created parcels based on the items-to-construct procedure (Little et al., 2002). Both correlational and structural equation analyses were conducted by means of the MPlus software package (Muthén and Muthén, 1998-2011). A robust maximum likelihood estimator (MLR) was used for all structural equation analyses, accounting for non-normality of the analyzed data (Muthén and Muthén, 1998-2011). Model fit was evaluated by means of a robust MLR χ^2^-test statistic, the Comparative Fit Index (CFI) and the Root Mean Square Error of Approximation (RMSEA), as well as the Standardized Root Mean Square Residual (SRMR). According to Hu and Bentler (1999), a CFI close to 0.95 indicates a good fit between the hypothesized model and the empirical data. For the RMSEA and SRMR values close to 0.06 and 0.08 are desirable, respectively. In order to determine the predictive power of the variables after controlling for prior SAS, we repeated structural equation analyses using residualized scores for wave 2 SAS controlling for age, global life satisfaction, and also wave 1 SAS.

Since we analyzed data of two waves (on average 2 years and 2 months apart), we had to face the challenge of missing data due to attrition, a common observation in longitudinal studies. Full-information maximum likelihood procedure (FIML) was used to handle missing data and estimate parameters as well as standard errors (Graham, 2009). Cases were excluded from analyses when they showed missing values in all variables constituting the prediction model. Since wave 2 participants differed slightly from wave 2 non-participants in their initial SAS-Content level (*d* = −0.36), we included auxiliary variables into our analyses, in order to reduce parameter estimation biases and loss of power (Graham, 2009). The consideration of auxiliary variables aims at reducing “the uncertainty caused by missing data and thereby improve the precision of the estimation” (Asparouhov and Muthén, 2008; p. 2). Auxiliary variables show associations to some of the variables in the structural model, but are not included into the model *per se* (Asparouhov and Muthén, 2008). Graham (2009) posited that especially the variables of interest but of a different wave constitute promising auxiliary variables, hence the first assessment of SAS at the beginning of the participants' studies was used as an auxiliary variable during the estimation process.

## Results

### Descriptive statistics and correlational analyses

Descriptive statistics of the raw data, as well as results of correlational and reliability analyses with residualized scores are displayed in Table 1. Regarding wave 2 SAS-Content, we observed significant correlations with several motivational variables: academic self-concept, achievement motivation, and subject-specific interest (intrinsic motivation for choosing teacher education). Furthermore, SAS-Content was related to neuroticism and conscientiousness, but showed no significant association to both cognitive (intelligence) and achievement-related variables (averaged grades in educational exams). SAS-Conditions was also associated with several motivational variables such as artistic and enterprising interests as well as educational interest, ability beliefs (both intrinsic motivations for choosing teacher education), and low difficulty of the studies (extrinsic motivation for choosing teacher education). Moreover, sex, averaged grades in educational exams, and neuroticism showed associations with this SAS dimension. In contrast to the large number of correlates observed for SAS-Content and SAS-Conditions, SAS-Coping was only significantly associated with neuroticism and sex.

**Table 1:** **Descriptive statistics, internal consistencies, and manifest correlations between potential predictor and criterion variables**.

	**Descriptive statistics**			**Correlations**		
	***M*^a^**	***SD*^a^**	**α^b^**	**Wave 2 SAS-Content^b^**	**Wave 2 SAS-Conditions^b^**	**Wave 2 SAS-Coping^b^**
**WAVE 2 SATISFACTION WITH THE ACADEMIC STUDIES**						
SAS-Content	3.92	0.72	0.83			
SAS-Conditions	2.72	0.84	0.80	0.24^***^		
SAS-Coping	3.29	0.97	0.81	0.34^***^	0.33^***^	
**DEMOGRAPHIC INFORMATION**						
Sex				0.07	0.19^**^	0.15^*^
Parental academic degree	2.75	1.01		−0.02	−0.01	0.06
**COGNITIVE AND ACHIEVEMENT-RELATED VARIABLES**						
Intelligence	26.15	2.93	0.75	−0.04	0.11	−0.01
Averaged grades	2.54	0.79		−0.06	−0.16^*^	−0.10
**MOTIVATIONAL VARIABLES**						
Vocational interest: realistic	2.43	0.77	0.85	−0.05	0.07	−0.01
Vocational interest: investigative	2.86	0.68	0.79	0.07	−0.04	−0.04
Vocational interest: artistic	3.32	0.82	0.84	0.09	−0.17^*^	−0.03
Vocational interest: social	3.83	0.63	0.86	0.12	−0.11	−0.05
Vocational interest: enterprising	3.43	0.62	0.81	0.11	−0.13^*^	0.06
Vocational interest: conventional	2.65	0.63	0.79	0.10	0.02	0.03
Academic self-concept	4.71	0.65	0.85	0.21^**^	0.02	0.05
Achievement Motivation	4.88	0.71	0.92	0.16^**^	−0.00	−0.08
Self-regulation	2.90	0.41	0.78	0.09	0.09	0.08
M: educational interest	3.57	0.47	0.90	0.09	−0.14^*^	−0.12
M: subject-specific interest	3.30	0.51	0.72	0.24^***^	0.05	−0.02
M: ability beliefs	3.32	0.48	0.79	0.09	−0.13^*^	−0.06
M: utility	3.00	0.53	0.86	0.03	−0.08	−0.09
M: social influences	2.33	0.68	0.81	0.10	−0.01	−0.07
M: low difficulty	1.36	0.50	0.81	−0.00	0.14^*^	0.11
**PERSONALITY**						
Neuroticism	2.69	0.62	0.85	−0.16^**^	−0.17^**^	−0.30^***^
Extraversion	3.57	0.47	0.77	0.09	−0.07	−0.03
Openness	3.36	0.52	0.72	0.09	−0.06	−0.01
Agreeableness	3.65	0.48	0.78	−0.00	0.10	−0.06
Conscientiousness	3.65	0.56	0.86	0.16^*^	0.10	−0.08

*M, mean; SD, standard deviation; SAS, Satisfaction with the academic studies; M, Motivation for choosing teacher education*.

a *Calculated based on raw scores*.

b *Calculated based on residualized values with age (and in case of wave 2 SAS variables also global life satisfaction) partialled out; Full information maximum likelihood procedure was used to estimate all values*.

*** p < 0.001;

** p < 0.01;

* *p < 0.05*.

Neither self-regulation nor the three personality factors extraversion, openness to experience, and agreeableness were associated with any of the SAS dimensions. The same was true for parents' highest academic degree and intelligence.

### Structural equation analyses

In order to determine the predictive validity of the variables under study, we conducted separate structural equation analyses for each SAS dimension. Only predictor variables showing significant manifest correlations *with the criterion variables* were included in the model (see Section Descriptive Statistics and Correlational Analyses). The same held for observed correlations *between predictor variables*: When we found significant manifest correlations, they were also specified on the latent level. Factor loadings ranged from moderate to high within all three prediction models (a complete listing of both the manifest correlation coefficients between potential predictor variables and all factor loadings is provided in the Supplementary Material). We repeated structural equation analyses controlling for wave 1 SAS within the criterion variables, in order to gain insight into the predictive validity of the variables independently from prior SAS.

#### Prediction of SAS-content

**Figure 1:**
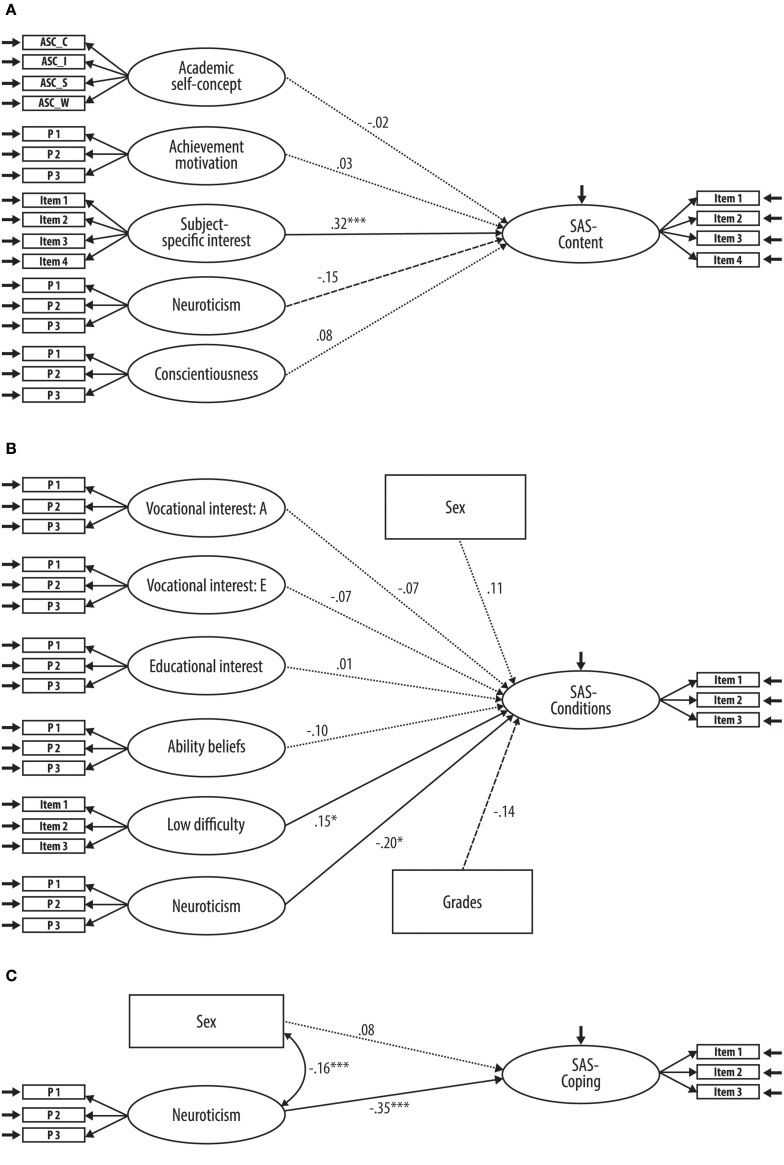
**(A–C)** Structural equation models to predict satisfaction with the content of the academic-program (SAS-Content; **A**), satisfaction with the terms and conditions of the academic program (SAS-Conditions; **B**), and satisfaction with the ability to cope with academic stress (SAS-Coping, **C**). All parameters were calculated based on residualized values with age (and in case of wave 2 SAS variables also global life satisfaction) partialled out. Full information maximum likelihood procedure was used to estimate all parameters. For the sake of a better readability, latent correlations between predictor variables are presented in Tables 2, 3. Standardized factor loadings are provided in the Supplementary Material. Full lines represent significant, dashed lines marginally significant, and dotted lines non-significant structural weights. Sex was coded 0 = female, 1 = male. ASC_S, academic self-concept: social reference; ASC_I, academic self-concept: individual reference; ASC_C, academic self-concept: criterion-oriented reference; ASC_W, academic self-concept without reference; A, Artistic; E, Enterprising; P, Parcel; *n* = 578–620. ^*^p < 0.05; ^***^p < 0.001.

Based on the results of the correlational analyses, academic self-concept, achievement motivation, subject-specific interest (intrinsic motivation for choosing teacher education), neuroticism, and conscientiousness were included as predictor variables into the model (see Figure 1A). The model yielded a good fit to the empirical data (χ^2^ = 345.60, *df* = 175, *p* < 0.001; CFI = 0.97; RMSEA = 0.04; SRMR = 0.05) and the predictors explained 15% of the total variance in SAS-Content. However, when all predictor variables were simultaneously considered in one model, only subject-specific interest (intrinsic motivation for choosing teacher education) correlated with subsequent SAS-Content (β = 0.32, *p* < 0.001). The association with neuroticism was only marginally significant (β = −0.15, *p* = 0.08). Latent correlations between the predictor variables are displayed in Table 2. We found similar results after prior SAS-Content has been taken into account. The model yielded a good fit to the data (χ^2^ = 343.75, *df* = 175, *p* < 0.001; CFI = 0.97; RMSEA = 0.04; SRMR = 0.05) and subject-specific interest served also as a significant predictor (β = 0.21, *p* = 0.03). Moreover, neuroticism was now significantly related to the criterion as well (β = −0.18, *p* = 0.04).

**Table 2:** **Latent correlations between predictor variables of SAS-Content**.

	**1**.	**2**.	**3**.	**4**.
**MOTIVATIONAL VARIABLES**				
1. Academic self-concept				
2. Achievement motivation	0.47^***^			
3. Motivation for choosing teacher education: subject-specific interest	0.33^***^	0.29^***^		
**PERSONALITY**				
4. Neuroticism	−0.31^***^	−0.24^***^	^a^	
5. Conscientiousness	0.26^***^	0.48^***^	0.18^**^	−0.25^***^

*Full information maximum likelihood procedure was used to estimate all values*.

a *Not included in the prediction model based on a non-significant manifest correlation*.

*** p < 0.001;

** *p < 0.01*.

#### Prediction of SAS-conditions

Artistic interest, enterprising interest, sex, neuroticism, and averaged grades in educational exams as well as different motivations for choosing teacher education (educational interest, ability beliefs, and low difficulty of the studies) were considered in the model to predict SAS-Conditions (see Figure 1B). Once again, the model fitted the empirical data well (χ^2^ = 323.32, *df* = 204, *p* < 0.001; CFI = 0.98; RMSEA = 0.03; SRMR = 0.04). The predictor variables accounted for 12% of the total variance in the criterion. When considering all variables simultaneously in one model, neuroticism (β = −0.20, *p* = 0.01) and the low difficulty of the studies (extrinsic motivation for choosing teacher education; β = 0.15, *p* = 0.03) significantly contributed to SAS-Conditions. The association between averaged grades in educational exams and the criterion was only marginally significant (β = −0.14, *p* = 0.09). Latent correlations between predictor variables are displayed in Table 3. Taking prior SAS-Conditions into account a similar pattern of results was found. The model again yielded a good fit to the data (χ^2^ = 322.40, *df* = 204, *p* < 0.001; CFI = 0.98; RMSEA = 0.03; SRMR = 0.04). Low difficulty of the studies (β = 0.17, *p* = 0.02) as well as neuroticism (β = −0.20, *p* = 0.02) showed significant associations with SAS-Conditions. Furthermore, averaged grades were now significantly related to the criterion variable (β = −0.19, *p* = 0.03).

**Table 3:** **Latent correlations between predictor variables of SAS-Conditions**.

	**1**.	**2**.	**3**.	**4**.	**5**.	**6**.	**7**.
**DEMOGRAPHIC INFORMATION**							
1. Sex							
**COGNITIVE AND ACHIEVEMENT-RELATED VARIABLES**							
2. Grades	^a^						
**MOTIVATIONAL VARIABLES**							
3. Vocational interest: artistic	−0.40^***^	^a^					
4. Vocational interest: enterprising	−0.08	^a^	0.28^***^				
5. Motivation for choosing teacher education: educational interest	−0.22^***^	^a^	0.31^***^	0.37^***^			
6. Motivation for choosing teacher education: ability beliefs	^a^	^a^	0.17^***^	0.45^***^	0.47^***^		
7. Motivation for choosing teacher education: low difficulty	0.14^***^	0.11^*^	^a^	−0.15^**^	−0.20^***^	−0.17^***^	
**PERSONALITY**							
8. Neuroticism	−0.15^***^	^a^	^a^	−0.20^***^	−0.12^*^	−0.24^***^	0.08

*Full information maximum likelihood procedure was used to estimate all values; Sex was coded 0 = female, 1 = male*.

a *Not included in the prediction model based on a non-significant manifest correlation*.

*** p < 0.001;

** p < 0.01;

* *p < 0.05*.

#### Prediction of SAS-coping

Regarding SAS-Coping, only sex and neuroticism had shown significant associations on the manifest level and were consequently part of the prediction model of this particular SAS dimension (see Figure 1C). The model provided an excellent fit to the data (χ^2^ = 3.97, *df* = 12, *p* = 0.98; CFI = 1.00; RMSEA = 0.00; SRMR = 0.01). In sum, the predictors explained 14% of the total variance in SAS-Coping. However, only neuroticism correlated with subsequent SAS-Coping significantly (β = −0.35, *p* < 0.001) when both variables were considered in one model. Taking prior SAS-Coping into account led to similar results. The model fitted the data excellently (χ^2^ = 3.05, *df* = 12, *p* = 1.00; CFI = 1.00; RMSEA = 0.00; SRMR = 0.02). Neuroticism was significantly associated with SAS (β = −0.26, *p* < 0.01), whereas the relation between sex and the criterion just failed to reach significance (β = 0.13, *p* = 0.06).

## Discussion

The purpose of our study was to determine how students' individual characteristics predicted the three SAS dimensions (SAS-Content, SAS-Conditions, SAS-Coping) assessed approximately 2 years apart. We focused on individual variables as predictors of SAS, although it may be argued that external circumstances (e.g., student's financial situation, housing) are of similar relevance. However, it rather is the personal evaluation component of these external circumstances that is substantially associated with someone's SAS and individual differences in this personal evaluation are affected by individual differences in underlying personality and motivational characteristics. This assumption is in line with Diener et al.'s (1999) position that “people react differently to the same circumstances, and they evaluate conditions based on their unique expectations, values, and previous experiences” (p. 277). In addition to personality and motivational variables, we also examined the predictive power of cognitive and achievement-related variables, since it has been shown that students' performance is also associated with their SAS (Apenburg, 1980).

### The predictive power of personality

While examining the predictive power of the potential determinants of the three SAS dimensions, we observed domain-general and domain-specific effects. In terms of personality, neuroticism predicted subsequent SAS (albeit its structural weight was only marginally significant for SAS-Content). Students initially showing higher levels of neuroticism were less satisfied with the conditions of their academic program as well as their ability to cope with academic stress about 2 years later (hypothesis 1). The importance of neuroticism as a predictor of SAS was supported by the finding that neuroticism was significantly associated with all three SAS-dimensions even after the initial SAS level has been taken into account (research question 1). These results are in line with findings of a meta-analysis on personality and job satisfaction (Judge et al., 2002), which demonstrated that job satisfaction correlated highest with neuroticism. Moreover and in line with our assumption, the predictive validity of neuroticism differed between the three SAS dimensions (hypothesis 4). Whereas neuroticism was only marginally associated with SAS-Content (but reached statistical significance after controlling for wave 1 SAS), it was more relevant for SAS-Conditions and especially SAS-Coping. We believe that this pattern of results may reflect an increasing saturation of the SAS dimensions with trait aspects: Whereas SAS-Content focused primarily on the personal evaluation of their academic program, SAS-Coping covered a more extensive area of the students' lives by also reflecting aspects of work-life balance. Considering broader aspects of the participants' lives may have resulted in a stronger association between neuroticism and this SAS dimension.

However, unlike results from Judge et al. (2002), none of the remaining four personality factors predicted subsequent SAS after controlling for global life satisfaction. Although conscientiousness was significantly correlated with SAS-Content on a manifest level (as expected), we found no substantial association when it was considered simultaneously with other predictors in the same model (hypothesis 2). This might be due to significant latent correlations between the included predictor variables (see Table 2). Thus, the shared influences of the determinants was mainly assigned to subject-specific interest and also—to some extent—neuroticism. Regarding the predictive power of extraversion for SAS, we extrapolated our hypothesis from research on global life satisfaction and job satisfaction, which had suggested that individuals scoring high on extraversion also tended to be happier with their jobs and their life *per se* (Diener et al., 1992; Judge et al., 2002). A better personality-environment-fit for extraverts has been discussed as a possible explanation for this finding, because extraverts feel more comfortable in social situations and this engagement in social interaction is in turn demanded by society as well as in professional life (see Pavot et al., 1990; Diener et al., 1999). Since we analyzed data of students enrolled in teacher education, thus individuals pursuing a professional occupation *primarily* characterized by social interactions (e.g., with colleagues, parents, and mainly students in class), a pronounced advantage of extraverts regarding SAS may be assumed. However, this assumption was not supported by our data, maybe because we covered the initial and middle stages of the participants' studies in our analyses, where extensive social interactions were not yet required. The students' experiences regarding social interactions with actual students in class were still limited to a few practical courses at the time of investigation. Hence, it seems as if extraverts do not experience a better personality-environment-fit at least during their studies at the university. However, this might change once the students start their professional life as teachers (hypothesis 3).

### The predictive power of motivational and achievement-related variables

Apart from the motivation for choosing teacher education, none of the motivational variables substantially predicted the SAS dimensions assessed approximately 2 years later (hypothesis 6). This might in part be related to the lower stability of motivational variables compared to personality characteristics. However, the initial motivation for choosing teacher education significantly predicted students' subsequent SAS also after additionally controlling for wave 1 SAS (research question 1), supporting findings by Hiemisch et al. (2005) who had already demonstrated the importance of individual reasons for study choice in the context of SAS. As expected, this motivational construct was mainly related with the two dimensions SAS-Content and SAS-Conditions. However, different types of motivation for choosing teacher education were of relevance for different SAS dimensions. Students reporting that they had chosen their major because of their interest in the specific subject (intrinsic motivation) were also more satisfied with the contents of the academic program. Participants scoring higher on extrinsic motivation (i.e., the assumed low difficulty of the studies) were also more satisfied (in this case with the conditions of their academic program). This seems to be counterintuitive at first, but might reflect fewer demands of rather extrinsic motivated students on the academic program provided by their university.

Concerning the predictive power of cognitive and achievement-related variables, correlational analyses revealed that students with higher academic achievement were also more satisfied with the conditions of their academic program (hypothesis 5). This result is in line with previous research by Nauta (2007). Nevertheless, structural equation analysis revealed that academic achievement did not predict SAS-Conditions significantly. These findings may be explained by the time lag between the two assessment occasions. In a meta-analysis on the relationship between job performance and job satisfaction, Judge et al. (2001) reported a lower average corrected correlational coefficient for longitudinal designs compared to cross-sectional studies. Furthermore, the operationalization of the three dimensions of SAS was not congruent with our measurement of academic achievement in terms of generality and specificity. However, in the context of the relationship between job satisfaction and job performance it has been discussed that a certain fit regarding measurement specificity of predictor and criterion variable was needed for a proper prediction of the criterion (Fisher, 1980). Whereas it seems highly probable that students took the entirety of their experiences at university into account while evaluating their SAS, the assessment of academic achievement covered only one facet of their studies, namely educational science. Since German teacher candidates are additionally enrolled in at least two different school subjects (e.g., Math, German, English, Biology), future research should operationalize academic achievement in a more general way, e.g., by forming a composite variable, including all fields of their studies. Moreover, since our investigation period covered only the first half of the participants' academic studies, the effect of achievement on SAS in later study stages needs to be determined (see also Section Limitations and Outlook). However, after controlling for prior SAS a significant association between averaged grades and SAS-Content was found (research question 1), maybe because the participants obtained their exam grades only after the first assessment had taken place, hence averaged grades could only affect wave 2 SAS but not initial SAS levels. In this context, controlling for wave 1 SAS may have “purified” the criterion, leading to a stronger correlation between the two constructs.

### Limitations and outlook

We analyzed data of a sample of students enrolled in teacher education. Hence, it may be argued that the results cannot be generalized to students enrolled in different majors. However, German teacher candidates are not only enrolled in educational studies, but also in at least two different school subjects as already mentioned in Section The Predictive Power of Motivational and Achievement-Related Variables. Hence, our sample was relatively heterogeneous after all. Moreover, we based our conclusions on data of a comparatively large sample. Nevertheless, future research may want to conduct similar analyses at different universities and institutions with students majoring in different subjects in order to test the generalizability of our results. This is of particular importance with regard to our results on predicting SAS after controlling for prior SAS, since no other study has addressed this matter so far.

Regarding the prediction of the three SAS dimensions, we considered a great variety of variables as potential predictors. Nevertheless, a considerable amount of variance in the SAS dimensions remained unexplained. One possible explanation for this finding is that we analyzed data from only one university. Hence, a restriction of range especially in the SAS dimensions could have depressed the observed associations, leading to smaller portions of explained variance. Again this issue can be addressed by analyzing additional data from several different universities. An alternative explanation for the small portions of explained variance might be the fact that we controlled for global life satisfaction. In order to determine how this approach affected our results we repeated our analyses using wave 2 SAS scores controlling only for age but not global life satisfaction or wave 1 SAS^1^. These analyses led to a similar pattern of results (regarding the relevance of select variables as predictors of SAS). Nonetheless, the effect sizes differed, leading to larger portions of explained variances. The predictive validity of neuroticism was more pronounced, especially for SAS-Content and SAS-Coping, whereas the predictive power of more study-specific constructs decreased (e.g., the effect of subject-specific interest on SAS-Content). Hence, controlling for global life satisfaction led us to a more precise evaluation of the predictive validity of the variables for the specific and to-the point variance in SAS.

Moreover, although we investigated the predictive validity of cognitive, achievement-oriented, motivational, and personality variables on SAS, we did not cover all possible predictor variables that may be of relevance for the three SAS dimensions. For instance, we did not determine the predictive validity of organizational variables (e.g., enrolled courses, curricular and extracurricular offerings, quality of teaching), which might be powerful predictors especially for SAS-Conditions. Moreover, it seems reasonable to assume that a good mental and physical health as well as an effective application of coping mechanism affects SAS-Coping positively. Future research should include these variables into the prediction model. Moreover, it should be investigated how the construct SAS *per se* as well as its relations to the predictor variables change over time.

## Conclusion

Our goal was to shed more light on the predispositions of SAS, an outcome variable of similar importance to students as job satisfaction to employees. However, the research field of SAS has often been neglected in past research. Controlling for global life satisfaction on SAS, we identified individual variables of first-year students that were associated with the satisfaction with the contents and conditions of their academic program as well as their ability to cope with academic stress. Simultaneously considering a wide range of potential predictors (cognitive, achievement-related, motivational, and personality variables) allowed us to integrate the select findings of previous studies and to overcome limitations of past research. We found that mainly neuroticism and the initial motivation for choosing their major predicted subsequent SAS substantially, indicating that students unsatisfied with their academic studies differ from their satisfied fellow students already at the beginning of their studies. Revealing a similar pattern of results after controlling for wave 1 SAS, corroborated the importance of these variables as predictors of SAS.

## Author contributions

FW, JK, and FS developed the study concept. All authors contributed to the study design. Testing and data collection were performed by FW, JK, and SR. FW performed the data analysis and interpretation in close cooperation with JK and FS. FW drafted the manuscript. All authors provided critical revisions and approved the final version of the manuscript for submission.

### Conflict of interest statement

The authors declare that the research was conducted in the absence of any commercial or financial relationships that could be construed as a potential conflict of interest.

## Footnotes

^1^The three models fitted the data well (SAS-Content: χ^2^ = 337.52, *df* = 175, *p* < 0.001; CFI = 0.97; RMSEA = 0.04; SRMR = 0.05; *R*^2^ = 0.29; SAS-Conditions: χ^2^ = 324.29, *df* = 204, *p* < 0.001; CFI = 0.98; RMSEA = 0.03; SRMR = 0.04; *R*^2^ = 0.13; SAS-Coping: χ^2^ = 3.29, *df* = 12, *p* = 0.99; CFI = 1.00; RMSEA = 0.00; SRMR = 0.01; *R*^2^ = 0.34). Significant predictors of SAS-Content (without controlling for global life satisfaction or wave 1 SAS): subject-specific interest (β = 0.23) and neuroticism (β = −0.40; all *p*'s < 0.01). Significant predictors of SAS-Conditions (without controlling for global life satisfaction or wave 1 SAS): Low difficulty of the studies (β = 0.14), neuroticism (β = −0.28), and averaged grades (β = −0.15: all *p*'s < 0.05). Significant predictors of SAS-Coping (without controlling for global life satisfaction or wave 1 SAS): neuroticism (β = −0.59, *p* < 0.001).
