# Supplementary material for: University Students' Satisfaction with their Academic Studies: Personality and Motivation Matter
Source: Front Psychol. 2016 Feb 16;7:55. doi: 10.3389/fpsyg.2016.00055 (PMC4754397; doi:10.3389/fpsyg.2016.00055)
Supplement: Supplementary file 1 [file Table1.docx]

Table

*Manifest correlations between potential predictor variables*

|  | 1. | 2. | 3. | 4. | 5. | 6. | 7. | 8. | 9. | 10. | 11. | 12. | 13. | 14. | 15. | 16. | 17. | | 18. | | 19. | | | 20. | | 21. | | 22. | | 23. | |  |  |
| --- | --- | --- | --- | --- | --- | --- | --- | --- | --- | --- | --- | --- | --- | --- | --- | --- | --- | --- | --- | --- | --- | --- | --- | --- | --- | --- | --- | --- | --- | --- | --- | --- | --- |
| *Demographic information* | | | | | | | | | | | | | | | | | | | | | | | | | | | | | | | | | |
| 1. Sex |  |  |  |  |  |  |  |  |  |  |  |  |  |  |  |  |  | |  | |  | | |  | |  | |  | |  | |  |  |
| 1. PAD | -.00 |  |  |  |  |  |  |  |  |  |  |  |  |  |  |  |  | |  | |  | | |  | |  | |  | |  | |  |  |
| *Cognitive and achievement-related variables* | | | | | | | | | | | | | | | | | | | | | | | | | | | | | | | | | |
| 1. INT | **.11** | .00 |  |  |  |  |  |  |  |  |  |  |  |  |  |  |  | |  | |  | | |  | |  | |  | |  | |  |  |
| 1. Grades | -.04 | **-.09** | **-.22** |  |  |  |  |  |  |  |  |  |  |  |  |  |  | |  | |  | | |  | |  | |  | |  | |  |  |
| *Motivational variables* | | | | | | | | | | | | | | | | | | | | | | | | | | | | | | | | | |
| 1. VI (R) | **.34** | .03 | **.13** | -.00 |  |  |  |  |  |  |  |  |  |  |  |  |  | |  | | |  | |  | |  | |  | |  | |  |  |
| 1. VI (I) | **.25** | .03 | **.14** | **-.11** | **.58** |  |  |  |  |  |  |  |  |  |  |  |  | |  | | |  | |  | |  | |  | |  | |  |  |
| 1. VI (A) | **-.38** | .06 | -.07 | .02 | **-.22** | -.07 |  |  |  |  |  |  |  |  |  |  |  | |  | | |  | |  | |  | |  | |  | |  |  |
| 1. VI (S) | **-.34** | .05 | **-.15** | .06 | **-.14** | .02 | **.47** |  |  |  |  |  |  |  |  |  |  | |  | | |  | |  | |  | |  | |  | |  |  |
| 1. VI (E) | **-.09** | .07 | -.08 | .03 | **.12** | **.15** | **.26** | **.53** | . |  |  |  |  |  |  |  | |  | |  | | |  | |  | |  | |  | |  | |  |
| 1. VI (C) | .04 | -.04 | .05 | .02 | **.32** | **.24** | **.10** | **.21** | **.49** |  |  |  |  |  |  |  | |  | |  | | |  | |  | |  | |  | |  | |  |
| 1. ASC | -.07 | .05 | .07 | -.01 | -.08 | .06 | **.20** | **.20** | **.20** | **.12** |  |  |  |  |  |  | |  | |  | | |  | |  | |  | |  | |  | |  |
| 1. AM | -.07 | .00 | .01 | -.07 | **.17** | **.28** | **.12** | **.25** | **.36** | **.28** | **.45** |  |  |  |  |  | |  | |  | | |  | |  | |  | |  | |  | |  |
| 1. REG | -.03 | .04 | .03 | **-.13** | **.15** | **.24** | .04 | **.17** | **.16** | **.20** | **.30** | **.43** |  |  |  |  | |  | |  | | |  | |  | |  | |  | |  | |  |
| 1. M (EI) | **-.23** | .08 | **-.15** | .06 | **-.09** | -.04 | **.27** | **.62** | **.35** | .08 | **.16** | **.27** | **.12** |  |  |  | |  | |  | | |  | |  | |  | |  | |  | |  |
| 1. M (SSI) | -.04 | .05 | .05 | -.07 | .03 | **.11** | **.21** | **.18** | **.13** | .04 | **.29** | **.27** | **.12** | **.23** |  |  | |  | |  | | |  | |  | |  | |  | |  | |  |
| 1. M (AB) | -.03 | **.08** | .07 | -.02 | .07 | **.20** | **.16** | **.25** | **.35** | **.23** | **.31** | **.36** | **.25** | **.39** | **.23** |  | |  | |  | | |  | |  | |  | |  | |  | |  |
| 1. M (U) | **.08** | **.08** | .04 | -.07 | .01 | -.06 | -.06 | **-.08** | .07 | .05 | .04 | .05 | -.02 | -.05 | .03 | **.08** | |  | |  | | |  | |  | |  | |  | |  | |  |
| 1. M (SI) | .04 | .05 | -.07 | .04 | -.01 | -.05 | .01 | .04 | .07 | **.09** | .04 | .07 | -.05 | .07 | **.15** | **.11** | | **.23** | |  | | |  | |  | |  | |  | |  | |  |
| 1. M (LD) | **.16** | -.02 | **-.10** | **.10** | .04 | -.05 | -.08 | **-.19** | **-.16** | -.01 | **-.15** | **-.24** | **-.17** | **-.19** | **-.08** | **-.16** | | .04 | | **.12** | | |  | |  | |  | |  | |  | |  |
| *Personality* | | | | | | | | | | | | | | | | | | | | | | | | | | | | | | | | | |
| 1. N | **-.16** | .00 | -.03 | -.01 | **-.12** | -.08 | .06 | -.06 | **-.16** | -.06 | **-.28** | **-.21** | **-.37** | **-.09** | -.08 | **-.16** | | -.03 | | .02 | | | **.09** | |  | |  | |  | |  | |  |
| 1. E | **-.13** | .04 | -.05 | .06 | **.10** | .05 | **.14** | **.38** | **.36** | .05 | **.18** | **.25** | **.13** | **.33** | **.15** | **.18** | | **.09** | | **.09** | | | **-.15** | | **-.41** | |  | |  | |  | |  |
| 1. O | -.07 | **.11** | .06 | **-.11** | .02 | **.30** | **.50** | **.27** | **.18** | -.03 | **.20** | **.13** | **.14** | **.13** | **.14** | **.18** | | **-.12** | | **-.12** | | | **-.15** | | .00 | | .08 | |  | |  | |  |
| 1. A | **-.18** | .04 | -.06 | -.06 | -.08 | -.03 | **.18** | **.36** | **.09** | .05 | **.10** | *.*08 | **.15** | **.30** | **.16** | **.09** | | -.03 | | -.00 | | | **-.12** | | **-.19** | | **.32** | | **.15** | |  | |  |
| 1. C | **-.13** | -.08 | .07 | **-.20** | .05 | **.09** | .06 | **.19** | **.19** | **.29** | **.26** | **.44** | **.47** | **.17** | **.17** | **.27** | | .00 | | .03 | | | **-.22** | | **-.22** | | **.13** | | .06 | | **.27** | |  |

*Note.* All coefficients were calculated based on residualized values with age partialled out; full information maximum likelihood procedure was used to estimate all values; significant correlations are marked in bold; PAD = Parental academic degree; INT = Intelligence; VI (R) = Vocational interest: realistic; VI (I) = Vocational interest: investigative; VI (A) = Vocational interest: artistic; VI (S) = Vocational interest: social; VI (E) = Vocational interest: enterprising; VI (C) = Vocational interest: conventional; ASC = Academic self-concept; AM = Achievement motivation; REG = self-regulation; M (EI) = Motivation for choosing teacher education: educational interest; M (SSI) = Motivation for choosing teacher education: subject-specific interest; M (AB) = Motivation for choosing teacher education: ability beliefs; M (U) = Motivation for choosing teacher education: utility; M (SI) = Motivation for choosing teacher education: social influences; M (LD) = Motivation for choosing teacher education: low difficulty; N = Neuroticism; E = Extraversion, O = Openness to experience; A = Agreeableness; C = Conscientiousness.
