# Supplementary material for: University Students' Satisfaction with their Academic Studies: Personality and Motivation Matter
Source: Front Psychol. 2016 Feb 16;7:55. doi: 10.3389/fpsyg.2016.00055 (PMC4754397; doi:10.3389/fpsyg.2016.00055)
Supplement: Supplementary file 2 [file Table2.docx]

Table

*Standardized factor loadings of the models wave 2 SAS-Content, wave 2 SAS-Conditions, and wave 2 SAS Coping*

|  | SAS-Content | | | | | | |  | | SAS-Conditions | | | | | | |  | | SAS-Coping | | |  |
| --- | --- | --- | --- | --- | --- | --- | --- | --- | --- | --- | --- | --- | --- | --- | --- | --- | --- | --- | --- | --- | --- | --- |
|  | SAS-Content | ASC | AM | M (SSI) | N | C |  | | SAS-Conditions | | VI (A) | VI (E) | M (EI) | M (AB) | M (LD) | N | |  | | SAS-Coping | N | |
| SAS-Content_1^a^ | .71 |  |  |  |  |  |  | |  | |  |  |  |  |  |  | |  | |  |  | |
| SAS-Content_2 | .80 |  |  |  |  |  |  | |  | |  |  |  |  |  |  | |  | |  |  | |
| SAS-Content_3 | .71 |  |  |  |  |  |  | |  | |  |  |  |  |  |  | |  | |  |  | |
| SAS-Content_4 | .78 |  |  |  |  |  |  | |  | |  |  |  |  |  |  | |  | |  |  | |
| ASC_C^a^ |  | .89 |  |  |  |  |  | |  | |  |  |  |  |  |  | |  | |  |  | |
| ASC_I |  | .49 |  |  |  |  |  | |  | |  |  |  |  |  |  | |  | |  |  | |
| ASC_S |  | .74 |  |  |  |  |  | |  | |  |  |  |  |  |  | |  | |  |  | |
| ASC_W |  | .94 |  |  |  |  |  | |  | |  |  |  |  |  |  | |  | |  |  | |
| AM_P_1^a^ |  |  | .92 |  |  |  |  | |  | |  |  |  |  |  |  | |  | |  |  | |
| AM_P_2 |  |  | .93 |  |  |  |  | |  | |  |  |  |  |  |  | |  | |  |  | |
| AM_P_3 |  |  | .86 |  |  |  |  | |  | |  |  |  |  |  |  | |  | |  |  | |
| M (SSI)_1^a^ |  |  |  | .52 |  |  |  | |  | |  |  |  |  |  |  | |  | |  |  | |
| M (SSI)_2 |  |  |  | .82 |  |  |  | |  | |  |  |  |  |  |  | |  | |  |  | |
| M (SSI)_3 |  |  |  | .75 |  |  |  | |  | |  |  |  |  |  |  | |  | |  |  | |
| M (SSI)_4 |  |  |  | .47 |  |  |  | |  | |  |  |  |  |  |  | |  | |  |  | |
| N_P_1^a^ |  |  |  |  | .78 |  |  | |  | |  |  |  |  |  | .78 | |  | |  | .78 | |
| N_P_2 |  |  |  |  | .87 |  |  | |  | |  |  |  |  |  | .87 | |  | |  | .87 | |
| N_P_3 |  |  |  |  | .81 |  |  | |  | |  |  |  |  |  | .81 | |  | |  | .82 | |
| C_P_1^a^ |  |  |  |  |  | .85 |  | |  | |  |  |  |  |  |  | |  | |  |  | |
| C_P_2 |  |  |  |  |  | .84 |  | |  | |  |  |  |  |  |  | |  | |  |  | |
| C_P_3 |  |  |  |  |  | .84 |  | |  | |  |  |  |  |  |  | |  | |  |  | |
| SAS-Conditions_1^a^ |  |  |  |  |  |  |  | | .74 | |  |  |  |  |  |  | |  | |  |  | |
| SAS-Conditions_2 |  |  |  |  |  |  |  | | .73 | |  |  |  |  |  |  | |  | |  |  | |
| SAS-Conditions_3 |  |  |  |  |  |  |  | | .82 | |  |  |  |  |  |  | |  | |  |  | |
| VI (A)_P_1^a^ |  |  |  |  |  |  |  | |  | | .81 |  |  |  |  |  | |  | |  |  | |
| VI (A)_P_2 |  |  |  |  |  |  |  | |  | | .86 |  |  |  |  |  | |  | |  |  | |
| VI (A)_P_3 |  |  |  |  |  |  |  | |  | | .62 |  |  |  |  |  | |  | |  |  | |
| VI (E)_P_1^a^ |  |  |  |  |  |  |  | |  | |  | .87 |  |  |  |  | |  | |  |  | |
| VI (E)_P_1 |  |  |  |  |  |  |  | |  | |  | .70 |  |  |  |  | |  | |  |  | |
| VI (E)_P_1 |  |  |  |  |  |  |  | |  | |  | .71 |  |  |  |  | |  | |  |  | |
| M (EI)_P_1^a^ |  |  |  |  |  |  |  | |  | |  |  | .89 |  |  |  | |  | |  |  | |
| M (EI)_P_2 |  |  |  |  |  |  |  | |  | |  |  | .85 |  |  |  | |  | |  |  | |
| M (EI)_P_3 |  |  |  |  |  |  |  | |  | |  |  | .87 |  |  |  | |  | |  |  | |
| M (AB)_P_1^a^ |  |  |  |  |  |  |  | |  | |  |  |  | .83 |  |  | |  | |  |  | |
| M (AB)_P_2 |  |  |  |  |  |  |  | |  | |  |  |  | .70 |  |  | |  | |  |  | |
| M (AB)_P_3 |  |  |  |  |  |  |  | |  | |  |  |  | .62 |  |  | |  | |  |  | |
| M (LD)_1^a^ |  |  |  |  |  |  |  | |  | |  |  |  |  | .56 |  | |  | |  |  | |
| M (LD)_2 |  |  |  |  |  |  |  | |  | |  |  |  |  | .89 |  | |  | |  |  | |
| M (LD)_3 |  |  |  |  |  |  |  | |  | |  |  |  |  | .87 |  | |  | |  |  | |
| SAS-Coping_1^a^ |  |  |  |  |  |  |  | |  | |  |  |  |  |  |  | |  | | .62 |  | |
| SAS-Coping_2 |  |  |  |  |  |  |  | |  | |  |  |  |  |  |  | |  | | .85 |  | |
| SAS-Coping_3 |  |  |  |  |  |  |  | |  | |  |  |  |  |  |  | |  | | .82 |  | |

*Note.* Factor loadings displayed for each prediction model separately; All parameters were calculated based on residualized values with age (and in case of wave 2 SAS variables also global life satisfaction) partialled out. Full information maximum likelihood procedure was used to estimate all parameters; All factor loadings are significant on a *p* < .001 level; SAS = Satisfaction with academic studies; ASC = Academic self-concept; ASC_S = academic self-concept: social reference; ASC_I = academic self-concept: individual reference; ASC_C = academic self-concept: criterion-oriented reference; ASC_W = academic self-concept without reference; AM = Achievement motivation; M (SSI) = Motivation for choosing teacher education: subject-specific interest; N = Neuroticism; C = Conscientiousness; VI (A) = Vocational interest: Artistic interest; VI (E) = Vocational interest: Enterprising interest; M (EI) = Motivation for choosing teacher education: educational interest; M (AB) = Motivation for choosing teacher education: ability beliefs; M (LD) = Motivation for choosing teacher education: low difficulty; P = Parcel

^a^ In the unstandardized model the loading has been fixed to 1 in order to determine the metric of the latent factor.
